# Detection of endogenous lipids in chicken feathers distinct from preen gland constituents

**DOI:** 10.1007/s00709-020-01544-7

**Published:** 2020-08-26

**Authors:** Viktoria Zeisler-Diehl, Eshrak Ali Ali Al-Khutabi, Gregor Kirfel, Lukas Schreiber, Gerhild van Echten-Deckert, Volker Herzog

**Affiliations:** 1 Institut für Zellbiologie, Ulrich-Haberland-Str. 61A, D-53121 Bonn, Germany; 2 LIMES-Institut für Membranbiologie und Lipidbiochemie, Universität Bonn, Gerhard-Domagk-Str. 1, D-53121 Bonn, Germany; 3 Ökophysiologie der Pflanzen, Institut für Zelluläre und Molekulare Botanik, Universität Bonn, Kirschallee 1, D-53115 Bonn, Germany

**Keywords:** Feather lipids, Osmium tetroxide, Thin layer chromatography, Gas chromatography, Cholesterol, Unsatured fatty acids

## Abstract

Bird feather lipids are usually attributed to the oily secretion product of the uropygial (preen) gland. We have observed, however, that feathers exhibit a strong reaction with osmium tetroxide (OsO_4_), even after treatment with detergents. This leads us to postulate the existence of endogenous feather lipids distinct from preen gland lipids. In order to substantiate our hypothesis, we investigated down feathers from a 1-day-old chicken as their uropgygial gland is not functionally active. The results confirmed the osmiophilic reaction, which was concentrated in the center of barbs and strongly reduced after lipid extraction. In these lipid extracts, we identified using thin layer chromatography, cholesterol, various ceramides, glycolipids, phospholipids, and fatty acids, which closely resembled the lipid composition of the water barrier in the chicken-cornified epidermal envelope. This composition is clearly distinct from chicken uropygeal gland secretion (UGS) known to consist of fatty alcohols as part of aliphatic monoester waxes and of free, predominantly saturated, fatty acids. A filter assay showed a strong reactivity between OsO_4_ and the fatty acids C18:1 and C18:2 and with feather lipid extracts, but not with UGS. These observations were confirmed by gas chromatography detecting unsaturated fatty acids including C18:1 and C18:2 as well as cholesterol exclusively in chicken feathers. Our results indicate that (1) endogenous lipids are detectable in chicken feathers and distinct from UGS and (2) in analogy to the morphogenesis of the cornified envelope of chicken feather lipids that may have derived from cellular feather-precursors, apparently enduring the specific cell death during developmental feather cornification.

**Electronic supplementary material:**

The online version of this article (10.1007/s00709-020-01544-7) contains supplementary material, which is available to authorized users.

## Introduction

Feathers are part of the defining traits of modern birds and structurally as well as compositionally unique integumentary appendages which serve a variety of distinct functions: they protect birds as well as their eggs and fledglings from cold temperatures and from water. Feathers in wings and tail play important roles in controlling flight and often form a colorful plumage thereby serving socio-sexual communication, e.g., for courtship display or for species recognition and they provide camouflage to protect against potential predators (Saino et al. 2013). Feathers combine light weight with an extraordinary durability and high physical and chemical resistance. In spite of this wide variety of fine structural and functionally distinct features, a general morphology of bird feathers includes a central rachis with the calamus and paired lateral arborizations in the form of barbs and barbules. The unbranched calamus is the most proximal part of the rachis. Mainly in flight feathers, all barbules are equipped with hooklets by which adjacent barbules interact, giving rise to the feathers’ flight effecting abilities. Although the different feather types vary considerably in their fine structure, they show a surprisingly uniform molecular composition mainly of a specific protein formerly considered as beta-keratin of the avian family, now named corneous beta-protein (CBP; Holthaus et al. 2018). The specific supramolecular arrangement of CBPs provides feathers with the necessary stiffness and the indispensible elasticity both of which are essential for the role of feathers in bird flight and the various other feather functions (Greenwold and Sawyer 2011).

All feathers display hydrophobic and water-repellent features which have been mainly attributed to the preening behavior of birds, i.e., the distribution of the oily product from the uropygial gland secretion (UGS) on the feather surfaces of birds plumage (Salibian and Montalti 2009). However, it is doubtful that the water-repellent function solely depends on the distribution of UGS on feathers, as surgical removal of the uropygial gland of ducks did not result in a diminished barrier function of feathers. It has also been shown that the removal of external lipids from a duck’s plumage did not affect the duck’s ability to swim (Fabricius 1959). Apparently, the external lipids and waxes derived from the uropygial gland are not the only means to form a water-resistant barrier in the birds’ plumage. When surfactants are added to the water, ducks are unable to swim and they drown (Choules et al. 1978). Obviously, the surface tension of water is essential which shows that the quality of bird feather hydrophobicity is also determined by non-lipid factors such as the specific diameter and the spacing of the network of barbs and barbules: water droplets on this network adopt large contact angles that provide water repellency (Rijke 1968). It should be mentioned that this hypothesis has been disputed as it may not account for some species, in particular some water birds (Elowson 1984).

The rationale of this study is based on the morphogenetic analogy between feathers and epidermis in birds: the cornified epidermis is known to provide the essential barrier function, i.e., the protection against loss of water and mechanical stress. The protection against loss of water has been mainly attributed to the synthesis of specific barrier relevant lipids which increase during cornification about hundred-fold (van Echten-Deckert et al. 2007). Programmed cell death mechanisms during cornification shape morphogenetically the bird epidermis (Saathoff et al. 2004) and feathers (Yu et al. 2002; Chang et al. 2004; Alibardi 2018). Lipids of the epidermis not only survive these morphogenetic cell death processes but they are inreased in content (van Echten-Deckert et al. 2007). As feather structures derive developmentally from the embryonic subperiderm (Alibardi et al. 2016) and undergo cornification in analogy to epidermal morphogenesis, their lipids may also survive the specific cell death during feather cornification. We, therefore, postulated the occurrence of such lipids which may be referred to as endogenous feather lipids as compared to the UGS lipids distributed on the feather surfaces during preening.

In this report, we describe in chicken feathers the identification of endogenous lipids, which exist independently of the oily content derived from UGS. First hints on the existence of endogenous lipids came from the treatment of various types of chicken feathers with OsO_4_, which is known to react in particular with unsaturated fatty acids (Belazi et al. 2009) and which stained the feathers’ deep dark brown. Thin layer chromatography indeed revealed in all chicken feather types the existence of such endogenous lipids consisting of cholesterol, ceramides, glyolipids, phospholipids, and fatty acids. Gas chromatographic analyses confirmed the presence of cholesterol, revealed a variety of fatty acids including C16:1 and the OsO_4_-reactive fatty acids C18:1 and C18:2, and showed the absence of these constituents in UGS. We have described before that a similar composition of lipids establishes the water barrier in the cornified epidermal envelope of chicken (van Echten-Deckert et al. 2007). At present, it is not excluded that the endogenous lipids are mere developmental relics from the cellular precursors of bird feathers. It seems, however, likely that the endogenous lipids contribute to the water repellency of bird feathers in analogy to the water barrier function of lipids in the cornified epidermal envelope.

## Materials and methods

Three male, 4 months old, juvenile chickens (*Gallus domesticus*) were obtained from Dr. Inga Tiemann (Institut für Tierwissenschaften, Universität Bonn). These chickens had been stunned using a low voltage electrical system, and when unconscious, killed by bleeding and frozen at − 20 °C. Fifty-five male 1-day-old chickens (*Gallus domesticus*), purchased from Frostfutter Center (Nordhorn, Germany), were used in all preparations. The 1-day-old chicken had been killed by controlled atmosphere stunning using carbon dioxide and immediately frozen afterwards at − 20 °C.

### Chicken feather preparation

Down feathers, about 2.5 cm in lenth, from 1-day-old chickens were removed from the skin by the use of anatomical tweezers. This was performed with caution to avoid compression of the selected feather. The feathers were screened by light microscopy for possible structural defects caused by this collection procedure and further processed shortly after removal. As in down feathers, the barbs are freely movable and in order to exclude that they fall apart, they were used without being cut into smaller pieces (except for biochemical purposes, see below). The much larger feathers from juvenile birds were cut into smaller pieces (about 2 cm in diameter).

All chicken feathers were rinsed prior to use for 3 min in 0.25% Triton X-100 in 100 mM Tris-HCl-buffer, pH 7.4, in order to remove potential impurities. The Triton X-100 cleaned feathers were rinsed in water and dried.

### Chemicals and reagents

Two percent OsO_4_ solutions in unbuffered water were prepared from solid OsO_4_ in ampules (Merck, Darmstadt, Germany). One hundred percent chloroform, 100% methanol, 100% acetic acid, 4M NaOH stock solution in H_2_O, 100% isopropanol, Triton X-100, and 100% acetone, analytical grade, were from Merck. Silica gel RP-18 glass plates were obtained from Merck.

### Solutions

#### Extraction mixture

Chloroform/methanol/H_2_O (20:10:2, v/v/v), chloroform/methanol/0.1 M KCl (6:96:94, v/v/v), 300 mM ammonium acetate, 200 mM ammonium acetate in methanol/H_2_O, chloroform/methanol (1:1, v/v) were freshly prepared prior to use. Cupric sulfate in aqueous phosphoric acid and amido black staining solution were from Merck.

### Morphological methods

For treatment with OsO_4_, down feathers were immersed in toto in 2% OsO_4_ in unbuffered water and kept in the dark at room temperature for 4 to 24 h. The feathers were rinsed in water and dried twice in 10 ml 100% acetone. As a control, feathers were immersed for 4 to 24 h at room temperature in lipid extraction medium (see above) prior to treatment with OsO_4_ and dried after a brief rinse in distilled water followed by immersion in acetone and drying on filter paper.

#### Semiplume, contour, and wing feathers from juvenile chickens

Feathers were collected from a freshly killed 4-month-old male chicken, and portions (about 2 cm in length) of such feathers were immersed in 2% OsO_4_ as described for down feathers. Some of the feather preparations were stained for 5 min in 0.1% amidoblack (w/v) in 25% (v/v) isopropanol and 10% (v/v) acetic acid, followed by a 1-min wash in 25% (v/v) isopropanol and 10% (v/v) acetic acid, and a final rinse in water. The feathers were dried on filter paper.

#### Documentation of macroscopical observations

Down feathers, semiplume, tail, and wing feathers were photographed or scanned: for photography, the feathers were placed on white paper; pictures were taken with a Panasonic Lumix G81 camera equipped with a LEICA DG Macro-Elmar 45-mm lens (Leica Camera AG, Am Leitz-Park 5, 35578 Wetzlar, Germany). Scanning was performed using a CanoScan 9950 F-Scanner at a resolution of 1200 dpi using a black background for native feathers or a white background for OsO_4_-treated feathers.

#### Light microscopy

After treatment with 2% OsO4, chicken feathers were rinsed in distilled water and mounted under glass cover slips using Mowiol 4-88 (Merck KGaA, Darmstadt, Germany). Light micrographs were taken using an Axio Imager Z1 Zeiss widefield microscope (Carl Zeiss Microscopy GmbH, Oberkochen, Germany) equipped with a Plan-Apochromat × 63.

#### Scanning electron microscpy

Chicken feathers were treated with 2% OsO4 as described above. After rinsining with distilled water, the feathers were dehydrated in 100% acetone for 30 min at room temperature: the dried samples were mounted on aluminum holders and sputter coated with 2 nm platinum/palladium using an HR 208 coating device (Cresington, Watford, UK). SEM was performed at an acceleration voltage of 3 kV using a Verios 460 L (FEI, Eindhoven, the Netherlands) equipped with a through-lens secondary electron detector.

### Cellulose filter assay

Lipid extracts (for preparation see below, Biochemical Methods) were concentrated about hundred-fold and dripped on a cellulose filter (Macherey and Nagel, Düren (Germany), Gütegrad MN 615, 0.16-mm thick, 7 cm in diameter). Similarly, UGS and the purified lipids C12, C18:1 and C18:2 were applied onto the filter. After evaporation of the solute (chloroform/methanol/ water, 20:10:2, by volume), the filter was immersed in a 0.2% unbuffered solution of OsO_4_ for 20 min at room temperature (in the dark). The filter was rinsed extensively in water and dried prior to photography.

### Biochemical methods

#### Homogenization of feather samples

**Fig. 1:**
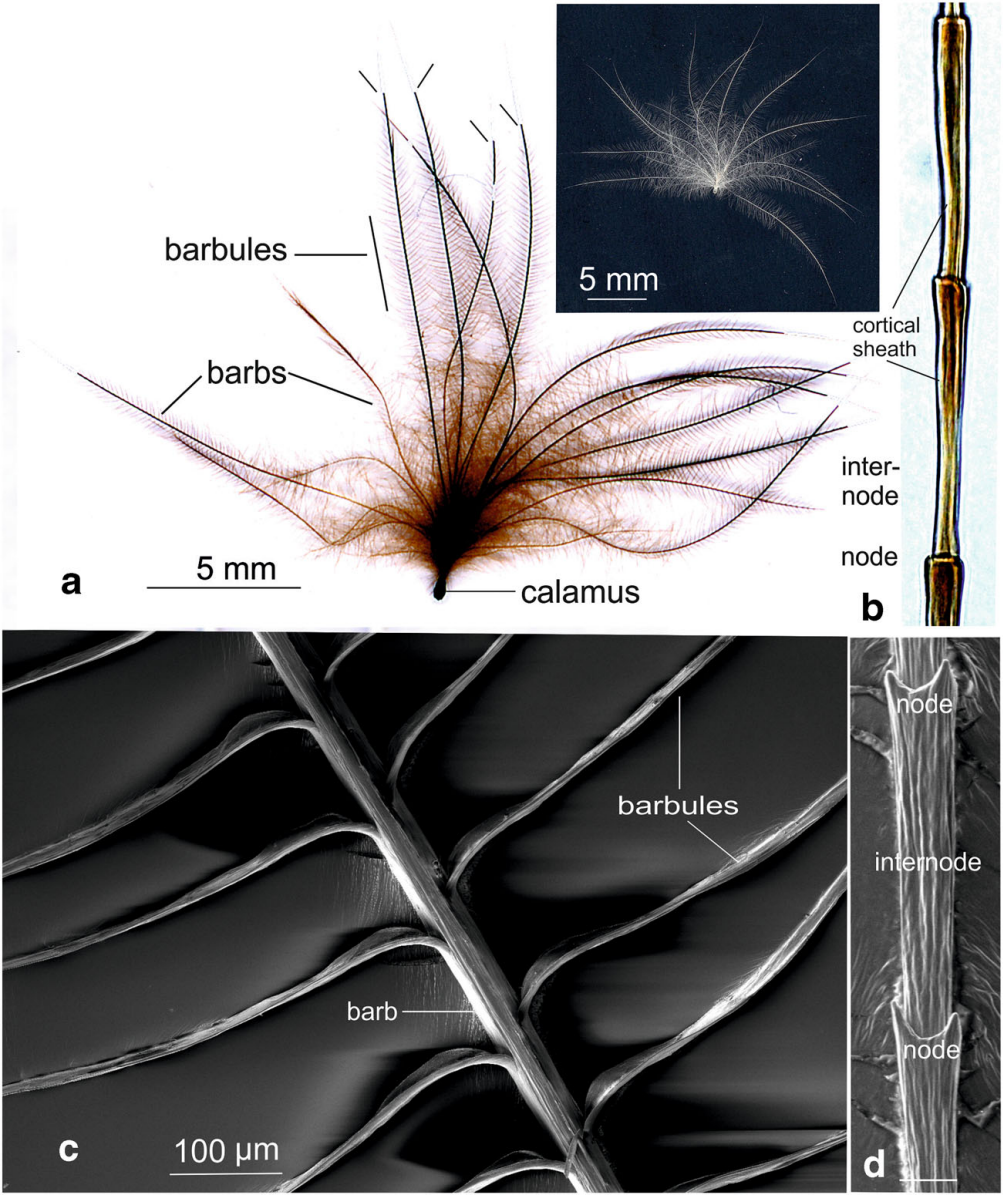
Down feathers from a 1-day-old chicken. Characteristically, all barbs originate from the very short rachis close to the calamus. This is in contrast to the semiplume down feathers from juvenile chicken (see Fig. 4). Treatment with OsO_4_ resulted in continuous osmium-black deposits in barbs, barbules, and the calamus region (**a**), located to the central portion of barbs and particularly concentrated in the node-areas (**b**). This centrally concentrated osmiophily was encased by the rigid corneous sheath (b) which appeared to remain unstained. Note that the very tips of the barbs occasionally lacked such osmium deposits. These always showed a sharp boundary between osmium-positive and –negative parts of the barbs (short lines). This lack of staining disappeared completely after prolonged treatment with OsO_4_, suggesting that the outer sheath of barbs may act as a diffusion barrier for OsO_4_. Scanning electron microscopy showed barbules and their origin from barbs (c). At higher magnification the substructure of barbule internodes and nodes as well as their spines became visible (d). Inset right: unstained down feather recorded against a black background

The calamus of each feather contains larger amounts of lipids due to the adherence of some dermal tissue (as recognized morphologically and shown in Fig. 1). Hence, for biochemical studies, the calamus of each feather was removed to avoid false positive results. The remaining feather parts were cut into small pieces with scissors. One hudred and fifty milligrams of feather were collected from each feather type using an analytical balance (Mettler Toledo™ NewClassic ME Analysenwaage, 53177 Bonn, Germany). Each of the preparations was homogenized in a 5-ml lipid extraction medium (chloroform/methanol/ water, 20:10:2, by volume) using an Ultraturrax Basic Homogenizer (Kinematika GmbH, Eschbach, Germany) at maximum speed (2.400 rpm) for 3 min, maintaing the temperature at 4 °C. The homogenates consisted mainly of feather particles of 1–5 μm in diameter and of a small fraction (< 5% as estimated from the area taken in electron micrgraphs), i.e., 2–3 barbule internodes (see Fig. S1 in ‚Supplementary Material’).

#### Lipid extraction

The feather homogenates were transferred into glass tubes closed with Teflon-lined screw caps, outside coated with parafilm, to avoid loss of the solvent by evaporation and placed in a shaking water bath for 24 to 48 h at 48 °C. The homogenates were filtered through glass wool–plugged Pasteur pipettes (the glass wool had been washed before twice in lipid extraction medium). The filtrates in the test tubes were dried under a stream of nitrogen. The specimens were kept for later use at − 20 °C. For determination of the total amount of lipid extracted from each feather type and from UGS, see below.

#### Thin layer chromatography

For the detection of ceramides, the dried lipids were dissolved in a 25-μl lipid extraction medium and applied to thin layer Silica gel 60 plates (TLC plates, Merck, Darmstadt, Germany). The TLC chamber was supplied with a 200-ml freshly prepared solvent (CHCL_3_/methanol/acetic acid 190:9:1, v/v/v). When the solvent mixture was 2 cm from top, the TLC plates were removed, dried, and placed above iodine granules. Finally, the plates were dipped in a solution of cupric sulfate in aqueous phosphoric acid and heated at 180 °C for 10 min (charcoaling).

#### Alkaline methanolysis of lipid extracts

The lipid samples were dissolved in 2.5 ml methanolic NaOH (100 μM) and placed in a shaking water bath for 2 h at 37 °C. To neutralize the samples, 10 μl of 100% acetic acid were added. Finally, the samples were dried under a stream of nitrogen and analyzed by TLC.

#### Identification and quantitative evaluation of lipids

Individual lipid bands were evaluated by photodensitometry (Shimadzu, Kyoto, Japan) and normalized to similar amounts of feathers determined by weighing.

### Gas chromatographic analysis of lipid extracts from feathers and UGS

The dried lipid extracts were dissolved in 5 ml chloroform:methanol (1:1 v/v). From these, an aliquot of 200 μl of each sample was transferred to clean analytical glasses. The organic solvent was dried under a gentle stream of nitrogen at 60 °C, and 1 ml of 1 N methanolic HCL (MeOH/HCL; Supelco) was added to each sample for transesterification. The samples were incubated for 2 h at 80 °C. After incubation, every sample was directly spiked with a 50-μl internal standard (C32 alkane, 10 mg/50 ml; Fluka), later enabling the quantification of the sample containing lipid compounds. Lipid monomers were subsequently extracted in hexane, and the total volume reduced to a final hexane volume of 200 μl. Free hydroxyl- and carboxyl groups being constituents of lipids were derivatized using the technique described recently (Baales et al. 2020, in press). In brief, derivatization was carried out by adding 20 μl BSTFA (N,O-bis (trimethylsilyl)-trifluoracetamid; Macherey-Nagel) and 20 μl pyridine (Sigma-Aldrích) to each sample. Derivatization took place in a heating block at 70 °C for 45 min. For quantification of the single compounds, 1 μl of each sample was injected on-column (30 m DB-1, inner diameter 0.32 mm, thickness 0.2 μm; J&W Scientific) to a gas chromatograph equipped with a flame ionization detector (GC-FID; GC- Hewlett-Packard 5890 series H). Identification of the single compounds was done by analyzing 1 μl of the samples by gas chromatography coupled to a mass spectrometer (GC-MS; quadrupole mass selective detector HP 5971, Hewlett-Packard) and by comparing the obtained fragmentation patterns with those stored in our homemade database or found in literature.

### Total amount of lipids in chicken feathers and in UG

#### Total amount of lipid as determined by gas chromatography

The dry weight of various types of chicken feathers from a 1-day-old and from a juvenile chicken was determined as desribed above and 150 mg of each sample were used. Likewise, 15 mg UGS were used for lipid extraction from UGS was performed. The lipid extracts were dried as described above and used for gas chromatographic determination of the specific lipid content in the various chicken feather types and in UGS. The total amount of lipid was expressed as mg per g feather or per g UGS.

## Results

The immersion of down feathers in 2% OsO_4_ known to react mainly with unsaturated lipids resulted in a strong staining of all structural components of chicken feathers. These morphological observations encouraged us to search biochemically for the presence of endogenous lipids and to analyze lipid extracts of feathers from a 1-day-old and juvenile chicken using thin layer and gas chromatographic techniques.

### Morphological indications for the presence of endogenous lipids in chicken feathers

Despite the remarkable structural differences in the various feather types from a 1-day-old and from juvenile chicken, we observed that all parts of the feathers exerted a strong reaction with OsO_4_.

### Down feathers from a 1-day-old chicken

Down feathers are recognized by their unique structural organization: in contrast to all other feather types (see Figs. 3, 4, and 5) all barbs originated from the almost punctiform short rachis. Photographs from down feathers after 2–4 h of OsO_4_ reaction showed deep brown deposits of osmium in all parts of the feather except for the occasional lack of staining in the most distal tips of the barbs (Fig. 1 a). Light microscope images revealed that the reaction of lipids with OsO_4_ was concentrated in the internal axis of barb internodes with the strongest reaction in the nodes (in Fig. 1 b). No reaction was observed on and within the cortical sheath encasing the axial osmiophilic region (Fig. 1b). However, it was also noted during OsO_4_ treatment of feathers, an almost immediate but weak OsO_4_ reaction suggesting the presence of lipids on the surface or within the cortical sheath. The occasional lack of OsO_4_ staining in the distal tips of barbs disappeared completely after prolonged treatment with OsO_4_ (4–6 h at room temperature). Hence, we consider this staining deficit at the tips of barbs due to the delayed staining probably caused by the outer protein sheath of barbs acting as a diffusion barrier for OsO_4_. Scanning electron microscopy showed barbules and their characteristic alternate origin from barbs (c in Fig. 1). Higher magnification revealed the substructure of barbule internodes and nodes and their spines (d in Fig. 1).

**Fig. 2:**
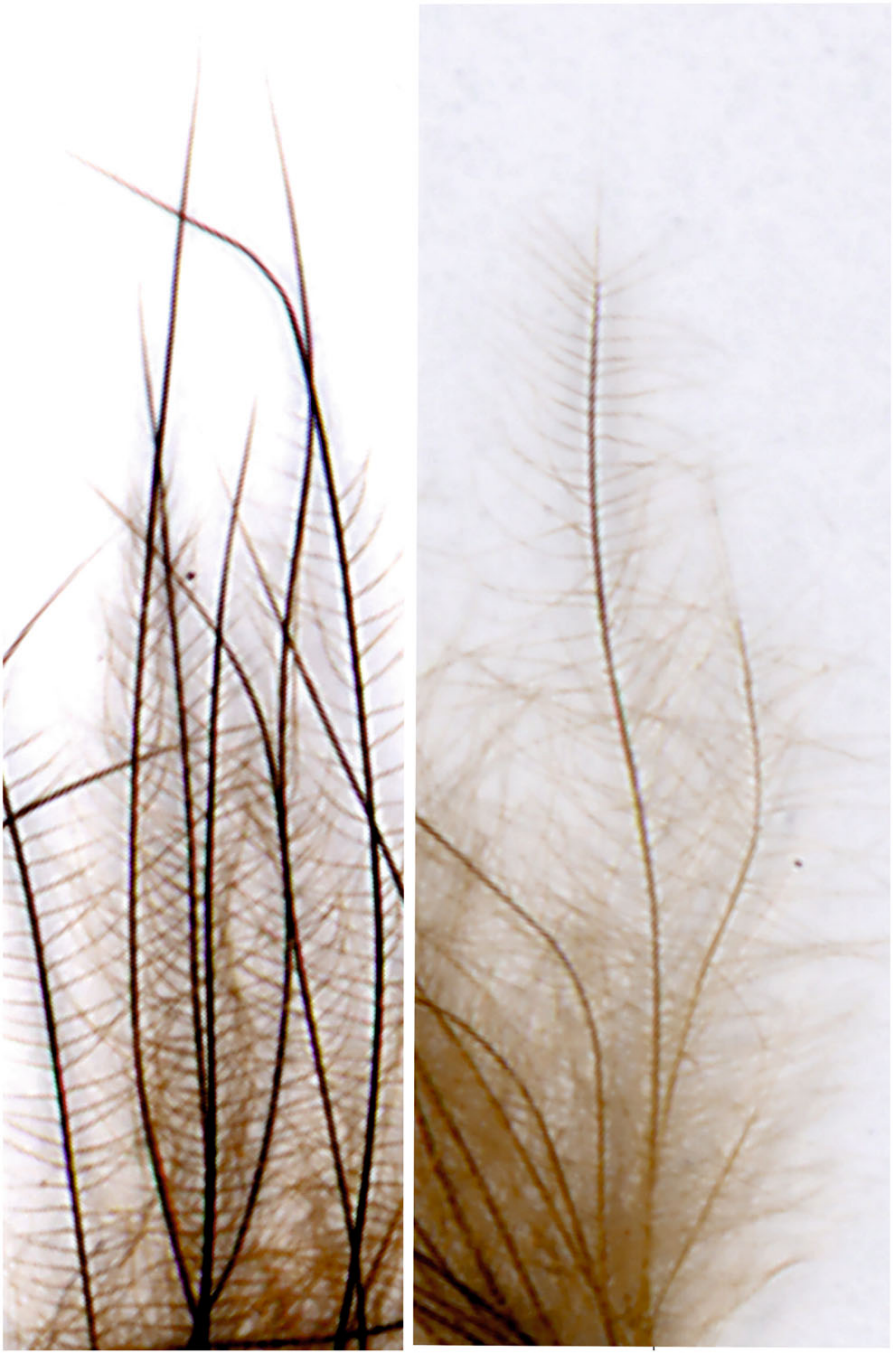
Down feather of a 1-day-old chicken without (left) and after (right) lipid extraction. Note the reduced osmiophily of feathers after lipid extraction

When feathers were immersed (24 h, 48 °C) in lipid extraction medium prior to the treatment with OsO_4_, a diminished OsO_4_-reactivity was observed, thus indicating the specificity of the reaction (Fig. 2), while also pointing out that after prolonged (4 days) lipid extraction a residual osmiophily remained recognizable. This residual osmiophilymay may be brought about by an incomplete lipids extraction.

### Flight, semiplume, and contour feathers from a juvenile chicken

**Fig. 3:**
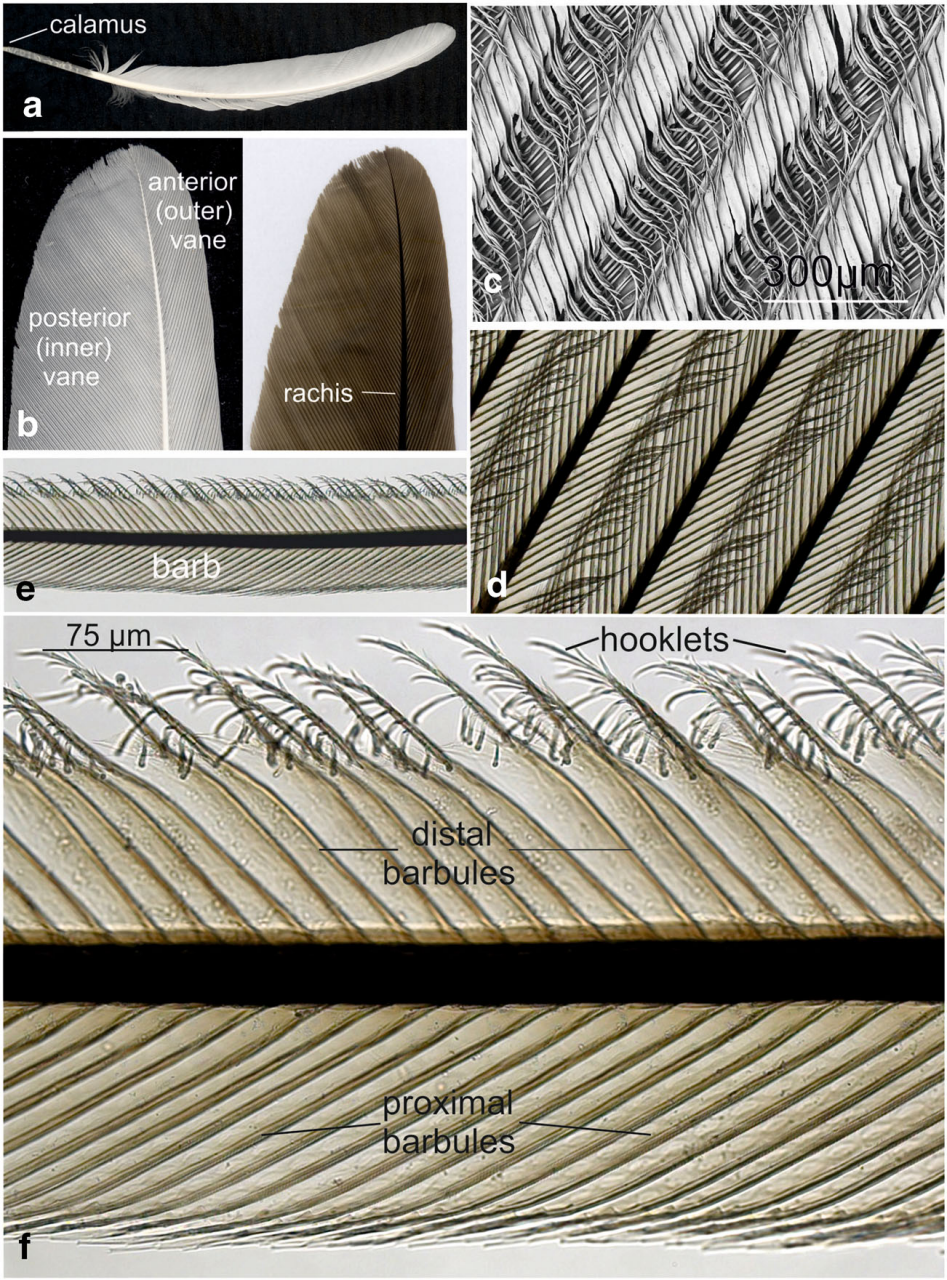
Flight feather from juvenile chicken. Native flight feather showing the characteristic gross anatomy with a broad posterior vane (**a**), the slender anterior vane and the central rachis with the calamus (**b**, left). Vanes and rachis reacted strongly with OsO4 (**b**, right). All structures were found to strongly interact with OsO_4_ (**d**, **e**, **f**). At higher magnification of single barbs (e), the details including the osmiophily of the hooklets on the distal barbules were recognized (**f**). Scanning electron microscopy revealed the carpet-like, repetitive vane structure of interacting barbs (c, view from top). Light micrographs at the same magnification showed the osmiophily of barbs viewed from underneath (d)

Flight feathers are stiff and asymmetrically shaped (Fig. 3). The primary function of flight feathers is to enable flight through the highly resistant vane. Its stability is mainly the result of strong interactions of the hooklets on the barbules with their hookless counterparts of neighboured barbs, thus forming a carpet-like network of the vane. All these parts of the flight feathers from a juvenile chicken reacted with OsO_4_ (Fig. 3, d–f). The carpet-like, repetitive vane structure of interacting barbs is shown by scanning electron microscopy (Fig. 3c, view from top). Light micrographs at the same magnification showed the osmiophily of barbs from underneath (Fig. 3 d). At higher magnification of single barbs (Fig. 3 e), the details including the osmiophily of the hooklets on the distal barbules were recognized (Fig. 3 f).

**Fig. 4:**
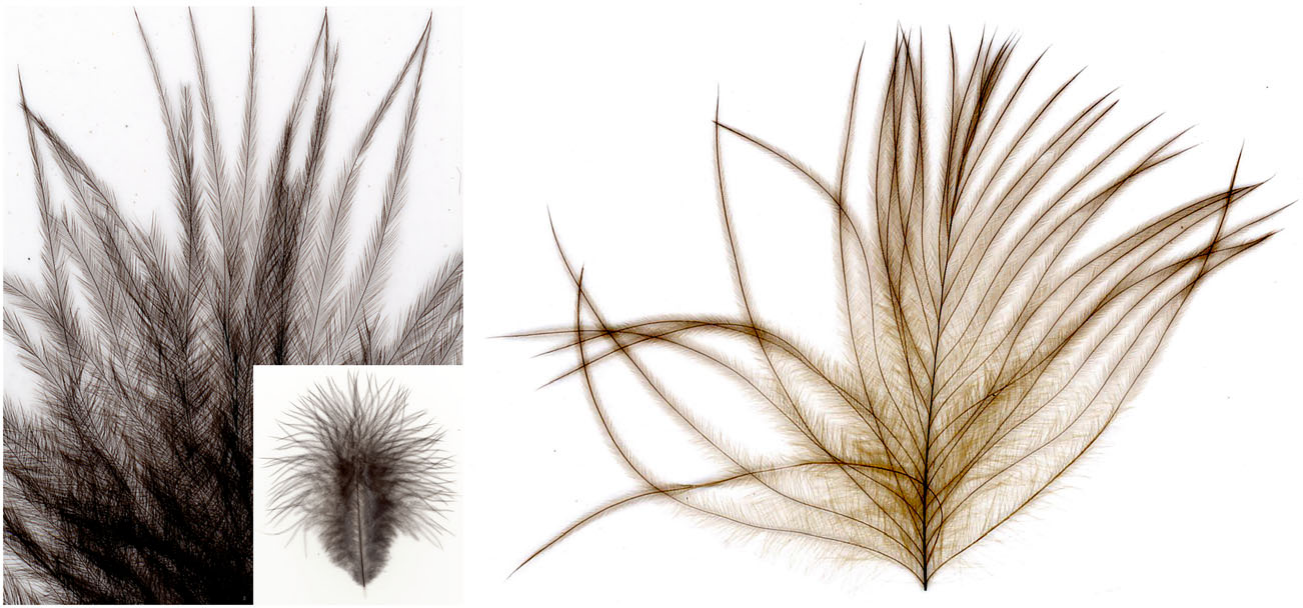
Semiplume down feather from a juvenile chicken after staining with amidoblack (left). Inset: all parts of the semiplume feather were stained thus visualizing the characteristic structure of this type of feather. In contrast to down feathers from a 1-day-old chicken, in semiplume feathers from a juvenile chicken the barbs branch off from the centrally located rachis. In semiplume feathers barbs do not interact with each other due to the lack of hooklets. After treatment with OsO_4_ (right), all parts of the feather were stained (due to the larger size of this feather type only its distal portion is shown whereas the calamus and other proximal parts have been removed)

In semiplume feathers from a juvenile chicken, the barbs originate, as in flight featrhers, from a centrally located rahis. In contrast to down feathers, the rachis spans the entire feather axis. Semiplume feathers from a juvenile chicken were stained with amidoblack in order to visualize their structural organization. The feathers were characteristically organized, showing an axial calamus, a centrally located rachis with most of the barbs branching off the rachis. In contrast to flight feathers (Fig. 3), their barbules were freely movable as their barbs did not interact with each other due to the lack of hooklets. In Fig. 4, these general morphological features are depicted in detail.

**Fig. 5:**
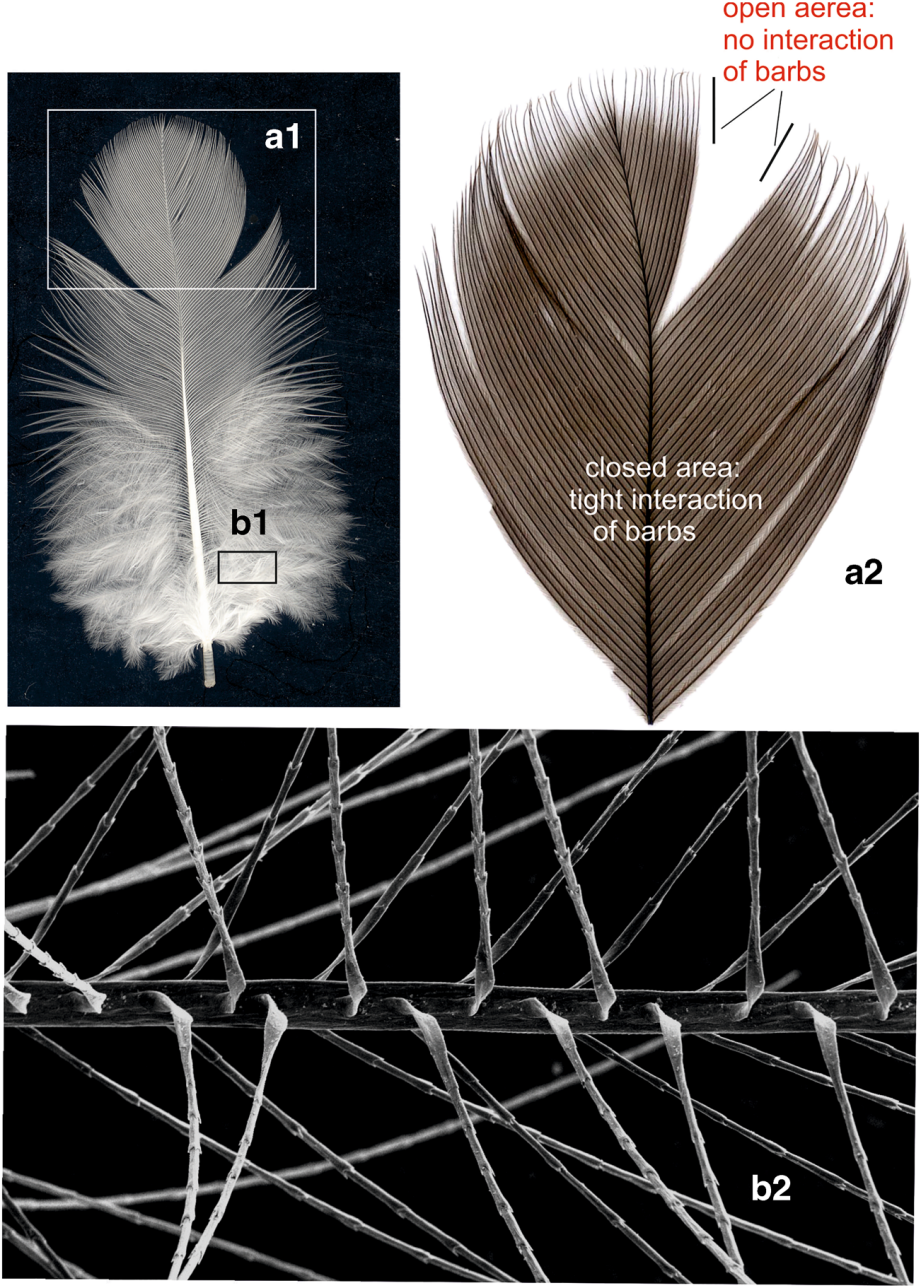
Contour feather of a juvenile chicken (A) with the proximal (A1) and the distal, plumulous part (B1). All parts of both feather types reacted strongly with OsO_4_ (A2, B2). Characteristically, hooklets are also present in contour feathers but located only in the central, closed area (A2) of tightly interacting barbs. The plumulous portion close to the basis of contour feathers was also osmiophilic (not shown) and consisted to a large part of barbs from which barbules extended into all four directions (B2)

Contour feathers (Fig. 5) form the major part of the plumage in most birds including chicken. In the distal portion of contour feathers, two major areas can be distinguished; the most distally located open portion in which the barbules show no interaction due to the lack of hooklets on their barbules. In the closed area, the barbs are characterized by their asymmetrically distributed hooks which enable a strong interaction with the adjacent barb. Characteristically of contour feathers, hooklets are also detectable but restricted to the central, closed area in which barbs interact tightly (Fig. 5A2). The hooklets become rare and disappear at the transition from the closed to the open area where no hooklets were detectable. An interesting detail was observed in the plumulous portion close to the basis of contour feathers: from the rachis osmiophilic barbules originate which extended into all four directions (Fig. 5B2).

### Filter assay for visualization of the reactivity of lipids and lipid extracts with osmium tetroxide

**Fig. 6:**
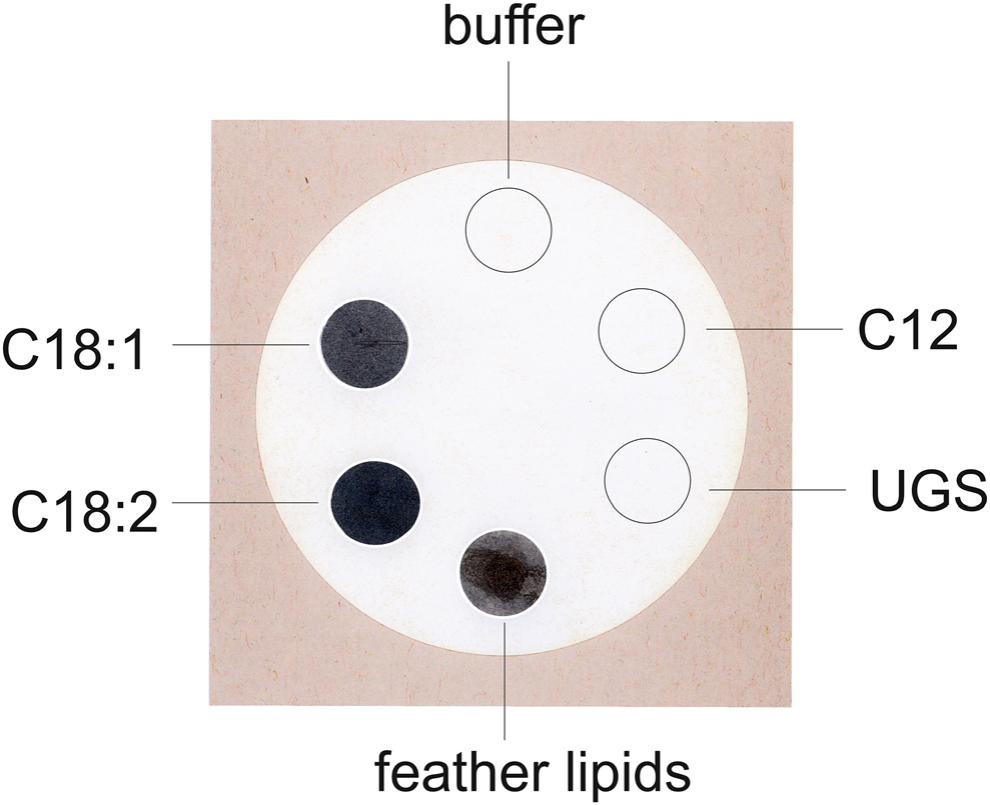
Purified fatty acids C12, C18:2, and C18:1 as well as feather lipid extracts and UGS were applied onto a cellulose filter and, after drying, immersed in a 0.2% OsO4 solution. Feather lipids and the unsaturated lipids C18:1 and C18:2 stained dark brown or black. In contrast, saturated fatty acids, C12, and UGS remained unstained. Buffer, negative control

The filter assay was deveolped as an intermediate between the morphological and biochemical studies. In this respect, the filter assay strongly supplemented the morphological data and gave hints which of the osmiophilic fatty acids could be expected in biochemical analyses. The filter assay revealed that the unsaturated lipds C18:1 and C18:2 and chicken lipid feather extracts react strongly with OsO_4_ (Fig. 6), whereas saturated lipids and the lipids extracted from UGS remained unreactive. However, the filter assay did not allow conclusions on the other lipids in feathers and in UGS.

### Biochemical analyses of the lipids in feathers and in UGS

#### Qualitative analysis of feather lipids

In a first approach, we assessed differences in the lipid content of down feathers and feathers from juvenile chicken.

**Fig. 7:**
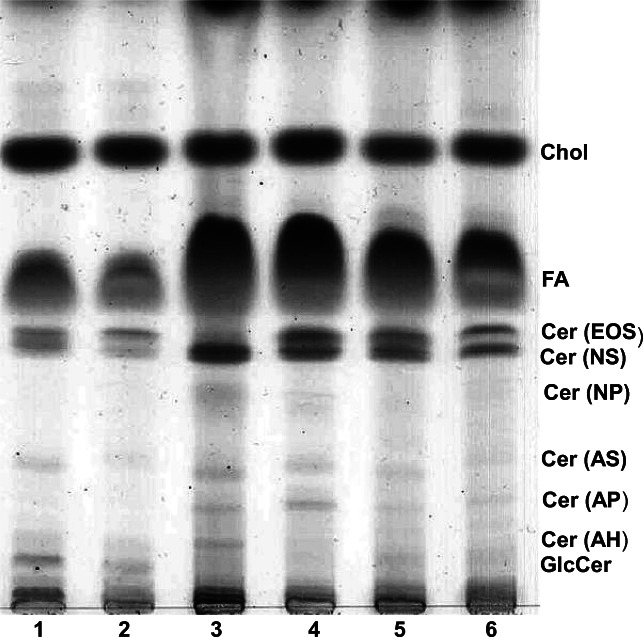
The effect of detergent pretreatment on the lipid profile of chicken feathers. Lipids of down feathers from a 1-day-old chicken (lanes 1 and 2) or of juvenile flight feathers (lanes 3 and 4) and contour feathers from the abdomen (lanes 5 and 6) were extracted and separated by TLC using chloroform/methanol/acetic acid (90:10:1, by volume) as solvent system. Prior to lipid extraction half of each feather sample was washed in the presence of Triton X-100 (0.25%) (lanes 2, 4, and 6), whereas the other half was not washed (lanes 1, 3, and 5). The abbreviations used are: Chol, cholesterol; FA, fatty acids, Cer, ceramide, GlcCer, glucosylceramide. The letters in the brackets denote cer structures and are explained in the text

**Fig. 8:**
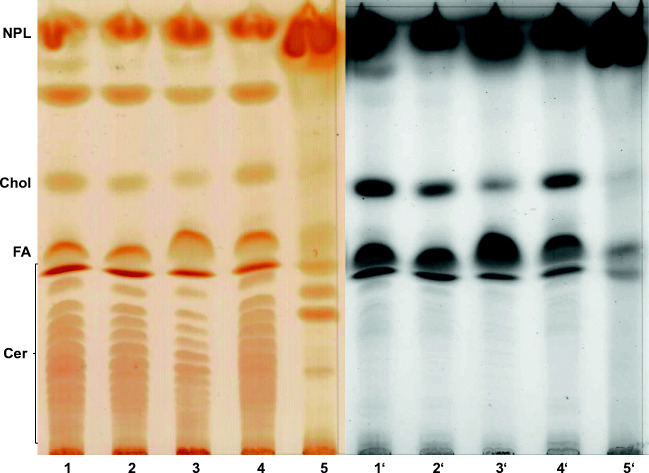
Lipids from feathers differ from those of the uropygial gland. Contour feathers (12 mg) from different domains of juvenile chicken, spine (lanes 1 and 1′), foot (lanes 2 and 2′), cloacal zone (lanes 3 and 3′), and feathers close to the uropygial gland (lanes 4 and 4′) were pretreated with 0.25% Triton X-100 and then extracted and separated by TLC with chloroform/methanol/ acetic acid (90:10:1, by volume) as mobile phase. Lipids from 1 ml of UGS (lanes 5 and 5′) were extracted and separated similarly. Lipids were visualized by iodine vapors (lanes 1–5) or by charcoaling (lanes 1′–5′). The abbreviations used are NPL, nonpolar lipids; chol, cholesterol; FA, fatty acids; Cer, different ceramide species

**Fig. 9:**
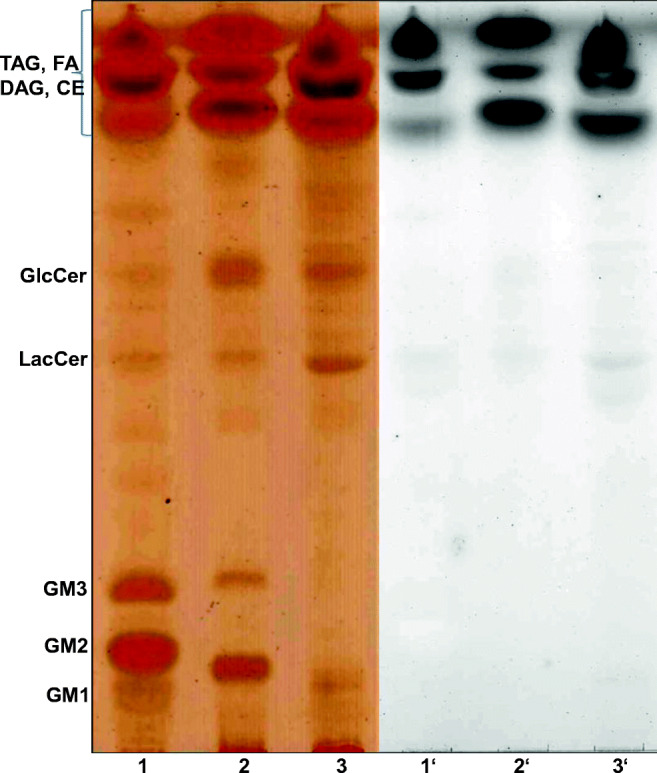
Lipid profiles of chicken feathers depend on the feather type and body domain. Down feathers (12 mg) from 1-day-old chicken (lanes 1 and 1′) and flight feathers (lanes 2 and 2′) or contour feathers of the abdomen (lanes 3 and 3′) from juvenile chicken were pretreated with 0.25% Triton X-100. Then, lipids were extracted, and subjected to mild alkaline treatment. Following reversed phase chromatography to delete small polar molecules, extracts were separated by TLC with chloroform/ methanol/ water (64:25:4, by volume) as mobile phase. Lipids were visualized by iodine vapors (lanes 1–3) or by charcoaling (lanes 1′–3′).The abbreviations used ar: CE, cholesterol ester; DAG, diacylglycerol; TAG, triacylglycerol; FA, fatty acids; GlcCer, glucosylceramide; LacCer, lactosylceramide. The terminology used for gangliosides GM3, GM2, and GM1 is that of Svennerholm (1963)

As shown in Fig. 7, feathers were subjected to different treatments prior to lipid extraction. Although rinsing the feathers in the presence of 0.25% Triton X-100 slightly reduces the total amount of lipids, we used this cleaning procedure since the overall lipid profile was not affected. The most prominent difference between feathers from a 1-day-old and juvenile chicken, respectively, is a considerable amount of GlcCer, which was present mainly in the former (Fig. 7, lanes 1 to 2). On the other hand, juvenile feathers, particularly flight feathers contained significantly higher amounts of free fatty acids as well as of certain ceramide species (Fig. 7, lanes 3 to 6). Note that in this study structural data of ceramides (Cer) cannot be provided but according to their Rf value might correspond to the epidermal Cer terminology proposed by Motta et al. (1993). Thus, Cer structures are defined by (i) their sphingoid base, either sphingosine (S), phytosphingosine (P), or 6-hydroxysphingosine (H); (ii) their amide bond fatty acid characterized by no hydroxyl group (N), an alpha-hydroxyl group (A), an omega-hydroxyl group (O), or an esterified omega-hydroxyl group (EO). Also, the amounts of nonpolar lipids including tri-and diacylglycerols, cholesterolesters and waxes, which are strongly accumulating in the front, are especially enriched in juvenile feathers, whereas the amount of cholesterol appears to be rather feather-type–independent. However, when juvenile feathers from different regions of the body were compared, the amount of cholesterol turned out to differ significantly (Fig. 8). Thus, contour feathers from the cloacal zone (Fig. 8, lane 3′) comprised the lowest amount of cholesterol but instead the highest content of free fatty acids suggesting that the total amount of lipids might be rather constant. Of interest, the uropygial gland secretion product also contained free fatty acids, ceramide, cholesterol, and nonpolar lipids, yet in a completely divergent ratio (Fig. 8 lanes 5 and 5′). The degree of unsaturation of lipids, as estimated by iodine visualization (Fig. 8, lanes 1–5), indicates that feather ceramides contain mainly unsaturated fatty acids. This result might explain why more complex sphingolipids were detectable by iodine vapors and hardly by charcoaling (Fig. 9). Visualization by iodine vapors reveals considerable differences between lipid composition of down feathers of 1-day-old chicken and juvenile feathers (Fig. 9). Although similar lipid species were detectable in all feathers analyzed, the amount of polar lipids (GM1, GM2, and GM3) was considerably higher in down feathers, whereas less polar lipids like GlcCer and LacCer were more abundant in juvenile feathers (Fig. 9).

In conclusion, ceramide composition shows no major differences between feathers from different areas of the juvenile chicken body (Figs. 7 and 8). Most importantly, the lipids from all feather types studied so far, even from feathers close to the uropygial gland, differ markedly from the composition of the gland’s lipid secretion product (Fig. 9).

#### Gas chromatographic analyses

In order to determine the total lipid content, to further specify the lipid composition of endogenous lipids and lipids from UGS and to search for the chemical basis of the osmium reaction in feathers, we analyzed the lipid extractions from all feather types by gas chromatography. The results show in general differences in the total content and clear differences in the composition and the quantity of various lipid species in the extracts of the various feather types and of UGS.

### Total content of endogenous lipids in chicken feathers and in UGS

The total amount of lipids results from the sum of all lipids detected in feathers and in UGS as determined by gas chromatography. We used the extracts of 150 mg from each feather type and of 15 mg from UGS. The total lipid content in chicken feathers was found at a mean of 9.0 ± 3.0 mg lipid per g feather and of 296 ± 40 mg lipid per g UGS. There were no significant differences in the total lipid content of down feathers from a 1-day-old chicken and of the various feather types from a juvenile chicken.

### Gaschromatographic spectra

**Fig. 10:**
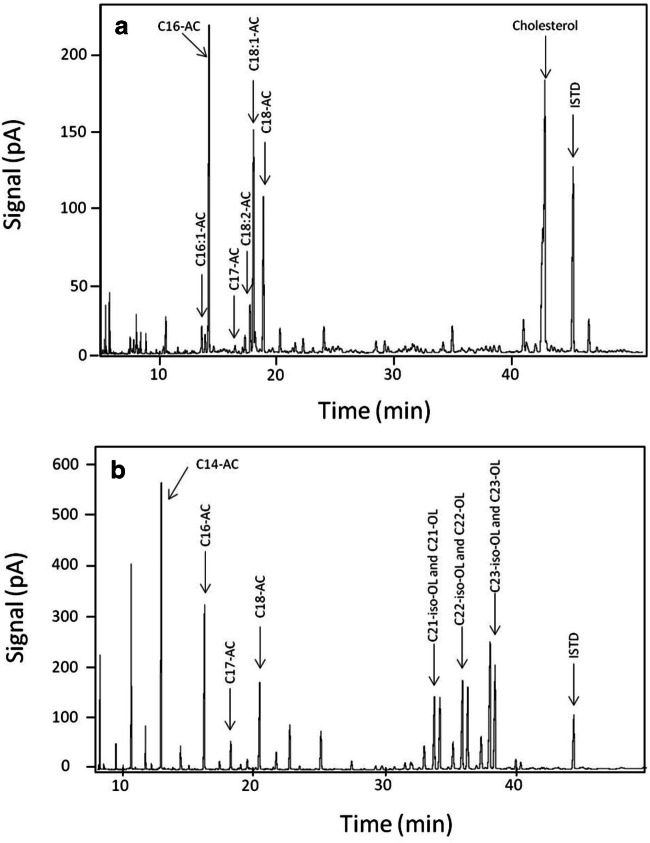
Chromatogram (GC-FID) of lipid monomers of down feathers of a 1-day-old chicken (**a**) and UGS (**b**) after transesterification using methanolic/HCl and derivatization with BSTFA. AC, acid; OL, alcohol; ISTD, internal standard

An overview on the lipids in chicken feathers is provided by the corresponding gas chromatographic spectra as shown in Fig. 10.

### Quantitative analyses of lipids in feathers and in UGS

**Fig. 11:**
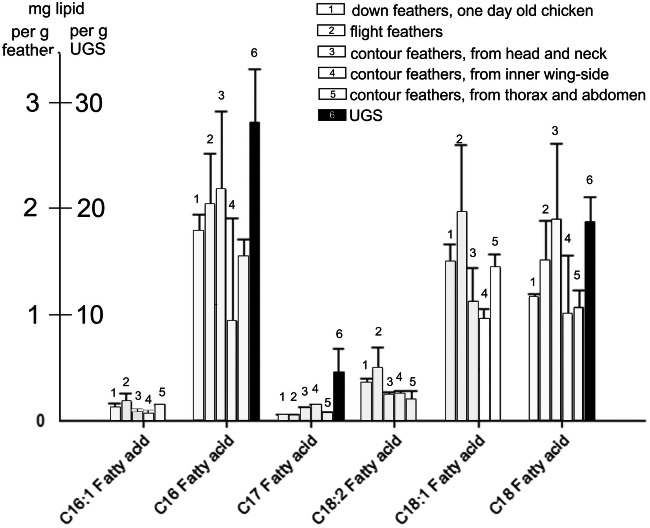
Overview of the most prominent fatty acids in lipids extracted from various types of chicken feathers and of UGS. The unsaturated fatty acids C16:1, C18:1, and C18:2 are the reaction partners for OsO_4_; they are present in all chicken feather extracts, but absent in UGS

Quantitative analysis of the lipid constitutents in various feather types and in UGS reavealed the siginificant differences in the content of cholesterol and of fatty acids, whereas cholesterol and the fatty acids C18:1 and C18.2 were found to be characteristic of feather lipids; these were absent in UGS. On the other hand, saturated fatty acids were found in both, UGS and feather lipids. Long-chain alcohols were restricted to UGS (not shown). Details of this finding can be seen in Fig. 11.

**Fig. 12:**
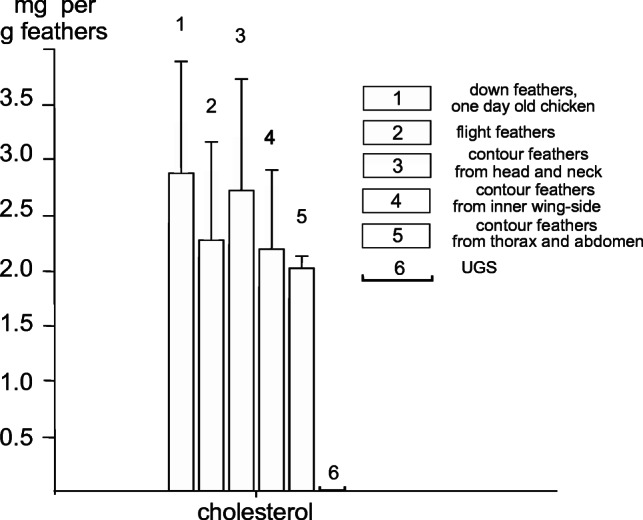
Cholesterol in various types of chicken feathers. Note that all feather types contain cholesterol, except of the UGS

Of particular interest is the distribution of cholesterol in chicken feathers and UGS extracts, it is found exclusively in chicken feather extracts but not detectable in UGS Fig. 12). The detection of cholesterol exclusively in feather lipid extracts reassured its distribution found by TLC (see Fig. 8). Furthermore, its distribution was found to be concordant to the distribution of unsaturated fatty acids as shown in Fig. 8. The mean values ± SD for all lipid constituents are shown in‚ Supplementary Material’ (Table S1).

## Discussion

In this manuscript, we have visualized and analyzed endogenous lipids which appear as regular constituents of chicken feathers. We refer to these lipids as “endogenous feather lipids”, thereby indicating that they are internal feather constituents as compared to the lipids in the uropygeal gland secretion (UGS) distributed exogenously onto the feathers during preening. We discuss the detection and composition of endogenous feather lipids, their distinction from the lipids of UGS as well as their possible significance for the specific functions of bird feathers, in particular their hydrophobicity.

### Morphological indications for the presence of lipids in chicken primary down feathers

Down feathers of a 1-day-old chicken differ structurally from feathers of a mature chicken. One major difference resides in the absence of the oily secretion product of the uropygial gland which is known not to be in a functional state yet at this early stage of post-hatch development (Romanoff 1960). We concluded firstly that the essential hydrophobicity of down feathers must be provided by other factors distinct from uropygial gland secretion (UGS) and secondly that lipids detected in chicken down feathers might instead be an endogenous feather constituent.

The morphological studies made use of OsO_4_ as a strong oxidant known to react with the double bonds of unsaturated fatty acids. The chemical basis of this cross-linking is the formation of stable diesters (Criegee et al. 1942), thus allowing the emergence of insoluble complexes. As osmium atoms are strongly light and electron dense, the lipids, e.g., in biological membranes, are not only fixed but simultaneously also stained by forming visible complexes thereby greatly enhancing the contrast, particularly in membranes, tissues, and cells (Wigglesworth 1957, 1988). Other cellular constituents such as undenatured proteins, nucleic acids, and carbohydrates do not react with OsO_4_ (Wigglesworth 1988). Hence, OsO_4_ has been considered to be a reliable histochemical reagent (Adams et al. 1967). More recently, observations with time-of-flight secondary ion mass spectrometry have shown that OsO_4_ colocalized with unsaturated C18 fatty acids and glyceride lipids containing these fatty acids (Belazi et al. 2009), thus supporting the earlier findings cited above. The treatment with OsO_4_ appears, therefore, a suitable method to visualize unsaturated lipids. In this study, such preparations provided the first hints that endogenous lipids are indeed contained in down feathers of a 1-day-old chicken and possibly in feathers of later developmental stages. The morphological observations on lipids in down feathers showed some peculiarities which require some attention:

1. In down feathers of a 1-day-old chicken, all parts including calamus, barbs, and barbules are stained dark brown upon treatment with OsO_4_. The osmiophily is confined to the inner zone of barbules and not detectable on the surface and within the cortical sheath of barbs. This, however, does not neccessarily imply that osmiophilic lipids are absent in this reagion. We still assume the presence of lipids within and on the cortical sheath as as we regularly observe a weak but almost immediate OsO_4_ reaction, whereas only after 2–4 h, the dense staining in the axial center becomes visible. In addition, the rinsing the feathers in the presence of 0.25% Triton X-100 slightly reduces the total amount of lipids (see Fig. 7), which can be explained by the surface localization of part of the lipids. Perhaps the lipids are masked and thus inaccessible for OsO_4_ due to the dense packaging of corneous protein molecules (see 2. below). Occasionally, the final tips of the barbules remained unstained (see Fig. 1). The staining, however, does become detectable after prolonged OsO_4_ treatment, suggesting a limited penetration ability of the cortical sheath (see Fig. 1b) for OsO_4_ and presumably the leakage of other, biologically relevant molecules, e.g., lipids from the matrix inside towards the outside.
2. After prolonged lipid extraction, the osmiophilic reaction of down feathers is strongly reduced, but a residual reactivity with OsO_4_ remains detectable, suggesting that some lipids within the feathers remain inaccessible for the extraction medium (see Fig. 2). This might be related to the bird feather CBPs: one of the leading features is the frequent occurrance of disulfide bridges that confer insolubility, strength, and rigidity by permanent, thermally stable cross-linking, resistant to most protein degrading enzymes (Lingham-Soliar et al. 2010). CBP molecules multimerise, thereby forming filaments consisting of multiple copies of the keratin monomer. In addition, hydrophobic interactions between apolar residues along the CBP helical segments represent a major force for the stability of the feather protein fiber (Hanukoglu and Ezra 2014). These features result in densely packed feather protein structures (Schweizer et al. 2006), thus forming, e.g., in the feather rachis (Lingham-Soliar et al. 2010) and the barb corneous sheath, a stable and rigid cortex. The formation of these cortical structures occurs during cell death and cornification of cortical cells, thereby enclosing lipids (Alibardi 2007; see final paragraph of “Discussion”). This densely packed corneous cortex may limit the penetrability of chicken feathers during the extraction of lipids, thus making understandable the residual osmiophily after lipid extraction of chicken feathers.
3. We found this osmiophily not only in all feather types of the chicken plumage but also in the feathers of other species such as peacock, pigeon, pheasant, and buzzard (unpublished), suggesting that the presence of endogenous lipids is probably a general feature of bird feathers, whereas the osmiophily allows only marginal conclusions on the underlying lipid species, thin layer chromatography provided insight into the composition of endogenous feather lipids in more detail.

### The composition of endogenous lipids in chicken feathers resembles the lipids in chicken epidermis

The endogenous lipids in all chicken feather types as analyzed by TLC contain prominent amounts of cholesterol, a variety of ceramides, phospholipids and free fatty acids. In this respect, endogenous feather lipids resemble at least in part the composition of cornified envelope-specific lipids. Specific ceramide species (Cer) which are known to constitute the lipid barrier of the epidermal stratum corneum (Wertz et al. 1985; Weerheim and Ponec 2001) were detectable in the various other chicken feather types as shown in Fig. 8. These Cer species have been reported to fulfill their function as the major diffusion barrier in the chicken epidermis (van Echten-Deckert et al. 2007). The chemical basis of this cornified envelope diffusion barrier might, in principle, also operate in chicken feathers in protecting the feathers from getting soaked and the skin surface of birds from coming into contact with cold water.

During epidermal development, the barrier forming lipids of the epidermis have their origin in the lipids of the subperiderm (van Echten-Deckert et al. 2007): the similarity between the lipids of feathers and of the subperiderm is in accordance with the hypothesis that bird feathers are developmentally related to the embryonic subperiderm (Sawyer et al. 2003; Alibardi et al. 2016), whereas the embryonic periderm is not involved in the formation of bird feathers. Similar to this finding, the localization of a histidine-rich epidermal differentiation protein has been interpreted to represent a developmental link between subperiderm and feather barbs and barbules (Alibardi et al. 2016). This developmental link has also been deduced from the expression of feather-type keratins in the subperiderm cells of the barb ridge lineages of feathers (Sawyer et al. 2003). Hence, the presence of these lipids may reflect the early stages of feather morphogenesis, and there are possibly developmental remnants which survived the specific cell death during the cornification of bird feathers.

The UGS product has been reported to contain aliphatic monoester waxes made of fatty acids and long-chain monohydroxy wax alcohols (Downing 1986). Here, we have shown that the endogenous feather lipids differ decisively in their composition from the oily secretion of the uropygial gland.

### Gas chromatographic studies revealed the presence of unsaturated C18 fatty acids: the basis for the reactivity of bird feathers with OsO_4_

In the filter assays, we have observed that the lipid extracts of feathers and the C18:1 and C18:2 fatty acids are osmiophilic, whereas saturated fatty acids and the extract of UGS remain unreactive (see Fig. 6). The gas chromatographic studies indeed revealed considerable amounts of C18:1 and C18:2 unsaturated fatty acids (see Table S1 in ‚Supplementary Material). They also showed the presence of cholesterol exclusively in chicken feathers. The saturated fatty acids C22–C26 were found in feather extracts only, whereas C15–C18 fatty acids were observed in both the extracts of feather lipids and of UGS. Glycerol and the long-chain alcohols C16, C17, and C19–24 were detectable in UGS only (Table 1).

**Table 1:** The major differences in the composition between chicken feathers and UGS as determined gas chromatographically

Constituents	In feather extract	in UGS extract
Cholesterol	Present	____________
C16:1, C18:1, C18:2 fatty acids	Present	____________
Fatty acids C22–C26	Present	____________
C15, C16, C17, and C18 fatty acids	Present	Present
Glycerol	____________	Present
Long-chain alcohols C16, C17, C19–24*	____________	Present

The differences in composition and concentrations of both, feather and UGS lipids, are shown in Figs. 11 and 12. The results for all lipid constituents are shown in Table S1 in‚ Supplementary Material

*Fatty alcohols as part of aliphatic monoester waxes

Hence, both unsaturated fatty acids (C18:1 and C18:2) form not only the basis for the osmiophily in the membranes of tissues and cells as generally used in electron microscopy (Belazi et al. 2009) but also of chicken feathers. The results confirm the endogenous nature of these feather lipids and exclude that their presence in chicken feathers might be caused by a contamination with the lipids from UGS.

Cholesterol is known to be synthesized by all cell types of the organism including keratinocytes of the birds’ epidermis. It takes a special position among the lipids studied so far as (1) cholesterol belongs to the constituents present in the chicken epidermis (Wertz et al. 1985; van Echten-Deckert et al. 2007) and, as reported here, in feathers but not in UGS; (2) cholesterol cannot be metabolized in these localizations; and (3) cholesterol cannot escape the central matrix of feathers due to the diffusion barrier the outer corneous sheath (see Fig. 1b). Cholesterol is usually released from the organism by the production of bile fluid as well as the activities of the kidney and the adrenal gland. Nevertheless, a small but significant amount of cholesterol is also released from the chicken organism by the loss of feathers, e.g., during molting. Interestingly, cholestanol, a sterol which is formed during reduction of cholesterol and normally found in bile fluid and intestine, has been detected also in the plumage of a variety of bird species (Bolliger and Varga 1961). This, however, does not necessarily imply that the reduction of cholesterol occurs in feathers. It might as well represent an intestinal contamination, e.g., during preening, since sterol could be removed from plumage by washing.

### Total amount of endogenous lipids in feathers and in UGS

With a total amount of 9 mg per g feather the lipid content in feathers is of course much lower than in UGS (about 260 mg per g UGS). However, according to the morphological data, the osmiophilic lipids appear evenly distributed in all chicken feather types and may thereby eventually exert their biological function effectively despite their low concentrations. In contrast, UGS on feathers was found (Kirfel and Herzog, in preparation) to be distributed in large clusters (about 1–5 μm) which were separated from each other by a distance of 5–10 μm (not shown).

### Endogenous lipids and feather hydrophobicity

Due to the findings reported here and in conjunction with observations of others, we conclude that at least three factors determine the hydrophobicity and contribute to the water-repellent function of chicken feathers:

1. The oily secretion of the uropygial gland: these lipids added onto the feather surfaces during preening consist of aliphatic monoester waxes, made of fatty acids and long-chain monohydroxy wax alcohols (Downing, 1986) and UGS has long been considered to be mainly responsible for the water repellency of bird feathers (Salibian and Montalti 2009).
2. Non-lipid factors: it has been shown that the hydrophobicity of bird feathers can in part be attributed to specific structural features, i.e., the diameter and the spacing of barbs and barbules. Several reports show that these features may lead to the “super-hydrophobicity” of bird feathers (Rijke 1968; Bormashenko et al. 2007; Srinivasan et al. 2014). Water droplets reside on feathers, i.e., the networks of barbs and barbules, partially on air pockets, thereby forming large contact angles that provide their water repellency. We have observed that prolonged extraction of endogenous lipids from down feathers of a 1-day-old chicken did not reduce the water repellency (unpublished). Therefore, their specific structure appears to be an additional factor leading to the hydrophobicity of down feathers (Kirfel and Herzog 2020 in preparation), thus confirming and emphasizing the significance of such non-lipid factors.
3. The endogenous feather lipids: as reported here the endogenous feather lipids consist of cholesterol, a variety of ceramide species, phospholipids, and free fatty acids. Thus, the speculation that endogenous lipids in chicken feathers may represent one of the factors determining the hydrophobicity of bird feathers appears to be obvious. However, we have as yet only indirect indications fort he presence of endogenous lipids on the surface and within the cortical sheath (see above, in “Discussion” of the “Morphological indications on the presence of lipids,” point 1). The study of these lipids in other bird species might be helpful, e.g., in the feathers of some aquatic birds such as cormorants: their feathers have been reported to be water permeant (Rijke 1968). As cormorants pursue their prey under water, their feathers are completely soaked by water and the resulting wetness of their feathers is the reason for the cormorant’s wing-spreading and feather-drying in the sun after diving (Rijke 1968; Marchant and Higgins 1990).

In conclusion, water repellency appears to be a vitally important function and perhaps multiple factors contribute to establishing and maintaining the hydrophobicity of bird feathers. The endogenous feather lipids described here may represent one of these factors. From the results in this work and from the work of others, a hypothesis on the origin of these lipids can be put forward: we have reported previously an about hundred-fold increase in the lipid content of keratinocytes during cornification which occurs during chicken development shortly before hatching (van Echten-Deckert et al. 2007). Due to their high lipid content, bird keratincytes have also been termed “sebokeratinocytes” (Menon and Menon 2000). It has been shown that an increase in the lipid content takes also place during feather morphogenesis (Alibardi 2007) and is named “lipidization” (Alibardi 2017, 2018). Apparently, lipidization is a characteristic function during the specific keratinocyte cell death and one of the key events during feather morphogenesis (Alibardi and Sawyer 2006). These lipids have beeen shown to accumulate among CBP bundles and to form a homogeneous lipid-protein matrix (Alibardi 2002). It is tempting to speculate that the endogenous feather lipids described in this report correspond to these lipds. However, as yet the composition of the lipids in the lipid-protein matrix mentioned above is unknown. Moreover, whereas the lipids in the descibed protein-lipid matrix are due to the formation and accumulation of lipid droplets (Alibardi 2007), the endogenous lipids as reported here consist of cholestrol and other membrane lipids, which do not occur in the form of lipid droplets. Hence, we postulate that endogenous lipids originate in the membranes of cells that have undergone cornification and cell death. Thereafter, these lipids may remain trapped in the mature feather and gain their surprisingly longlasting and stable features for at least three reasons: (1) Feather lipids cannot be removed as the blood vessels have vanished. Cholesterol, for example, normally metabolized in liver, kidney, and adrenal gland tissues (see above) is unable to gain access to these tissues and remains trapped within the feather. (2) Endogenous feather lipids cannot be metabolized as keratinocytes have undergone cornification and cell death. (3) Endogenous feather lipids are concentrated in the center, e.g., of barbs, and surrounded by the strong diffusion barrier provided by the CBP cortical sheath. As the lipids in the endogenous feather lipds as reported here correspond to the lipids in keratinocytes which increased so strongly during cornification (van Echten-Deckert et al. 2007), they may represent mere developmental relics of these cellular processes. Although we cannot exclude this possibility, it seems worthwhile in future work to search and to test for their involvement in water repellency and other properties of feathers.

## Electronic supplementary material

ESM 1 (DOCX 3990 kb)

ESM 2 (DOCX 3724 kb)
