# Supplementary material for: Detection of endogenous lipids in chicken feathers distinct from preen gland constituents
Source: Protoplasma. 2020 Aug 26;257(6):1709–24. doi: 10.1007/s00709-020-01544-7 (PMC7567736; doi:10.1007/s00709-020-01544-7)
Supplement: Supplementary file 1 — (DOCX 3990 kb) [file 709_2020_1544_MOESM1_ESM.docx]

**
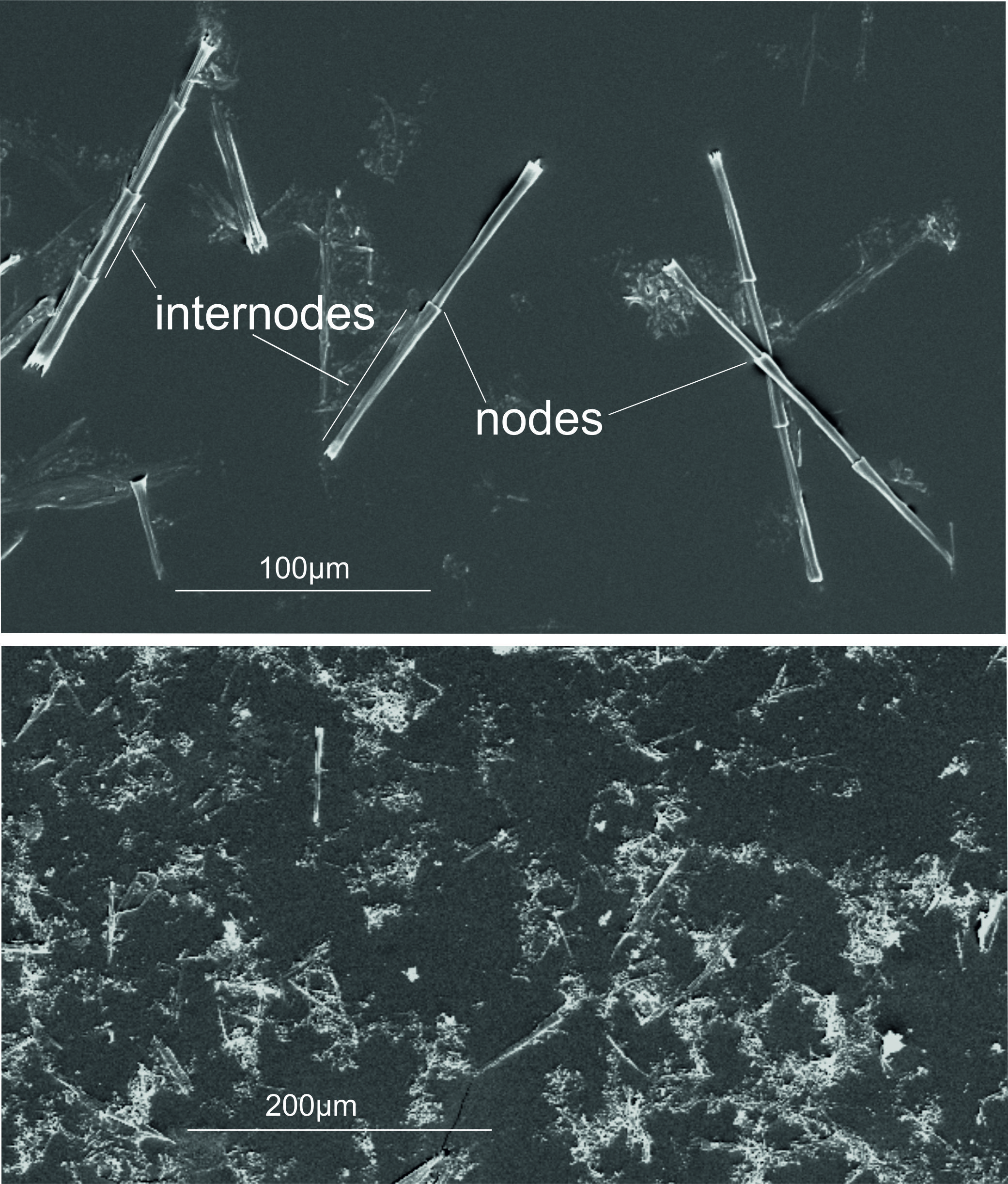
**

**Fig. S 1** Fractions of homogenized down feathers used for lipid extraction. Down feathers from one-day-old chicken were homomogenized as described. After lipid extraction the homogenate was filtered and the filtrate

was used for analysis by TLC or gas chromatography. The fractionated feathers remaining on the filter were

resuspended and prepared for scanning electron microscopic visualization, which revealed two components of different size and composition: top; a very minor fraction of filament-like constituents composed of 2-3 feather internodes (length 150 to 200µm; compare with Fig.1). These internodes derived from down feather barbs, their presence showed their high durability against the shearing forces during the homogenization process - Bottom: The much larger fraktion-part consisted of particles of different size (1-5µm) with a tendency to form aggregates.
