# Supplementary material for: Detection of endogenous lipids in chicken feathers distinct from preen gland constituents
Source: Protoplasma. 2020 Aug 26;257(6):1709–24. doi: 10.1007/s00709-020-01544-7 (PMC7567736; doi:10.1007/s00709-020-01544-7)
Supplement: Supplementary file 2 — (DOCX 3724 kb) [file 709_2020_1544_MOESM2_ESM.docx]

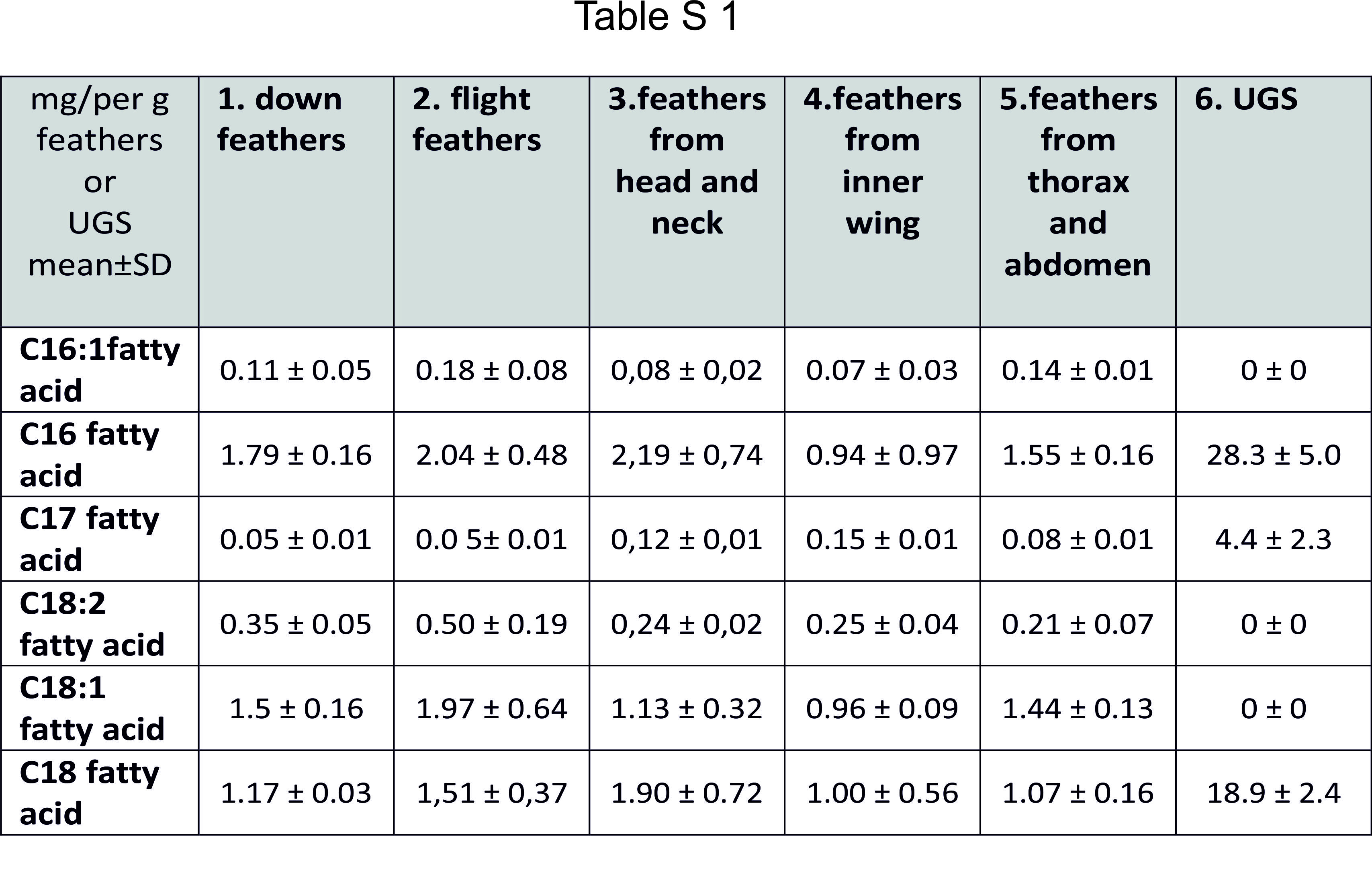


Quantitation of the main lipids from 3 feather- and UGS- determinations displaying the differences in the composition of endogenous feather lipids and of lipids in UGS.
